# The structure of Zika virus NS5 reveals a conserved domain conformation

**DOI:** 10.1038/ncomms14763

**Published:** 2017-03-27

**Authors:** Boxiao Wang, Xiao-Feng Tan, Stephanie Thurmond, Zhi-Min Zhang, Asher Lin, Rong Hai, Jikui Song

**Affiliations:** 1 Department of Biochemistry, University of California, Riverside, Riverside, California 92521, USA; 2 Department of Plant Pathology and Microbiology, University of California, Riverside, Riverside, California 92521, USA

## Abstract

The recent outbreak of Zika virus (ZIKV) has imposed a serious threat to public health. Here we report the crystal structure of the ZIKV NS5 protein in complex with *S*-adenosyl-L-homocysteine, in which the tandem methyltransferase (MTase) and RNA-dependent RNA polymerase (RdRp) domains stack into one of the two alternative conformations of flavivirus NS5 proteins. The activity of this NS5 protein is verified through a *de novo* RdRp assay on a subgenomic ZIKV RNA template. Importantly, our structural analysis leads to the identification of a potential drug-binding site of ZIKV NS5, which might facilitate the development of novel antivirals for ZIKV.

## Abstract (web summary)

The recent outbreak of Zika virus is a major worldwide health concern and effective drugs are currently not available. Here the authors present the structure of Zika non-structural protein 5 and identify a potential drug-binding site, which might facilitate the development of novel antivirals.

The outbreak of Zika virus (ZIKV) in the Americas and West Pacific islands in the past year has become a worldwide health concern, affecting more than 60 countries to date^1^,^2^. Increasing evidence has linked ZIKV infection to microcephaly in newborn infants^3^, and to Guillain–Barré syndrome in adults^4^. The fact that no vaccines or therapeutics for prevention or treatment of ZIKV infection are currently available further deepens the concern. To develop effective antivirals against ZIKV infection, it is urgent to gain a comprehensive structural and mechanistic understanding of the molecular machineries underpinning the life cycle of ZIKV.

ZIKV belongs to the family of flavivirus that includes a variety of mosquito-borne human pathogens, such as dengue virus (DENV1–4), yellow fever virus, West Nile virus, Spondweni virus and Japanese encephalitis virus (JEV)^5^. The genome of ZIKV is organized into the form of a single positive strand RNA, encoding in total three structural proteins (C, prM/M and E) and seven non-structural (NS) proteins (NS1, NS2A, NS2B, NS3, NS4A, NS4B and NS5)^6^. Among these, NS5 is the largest NS protein, containing an N-terminal methyltransferase (MTase) domain responsible for viral RNA capping and a C-terminal RNA-dependent RNA polymerase (RdRp) domain for viral RNA synthesis, with evidence indicating that the MTase and RdRp domains cooperate in RNA synthesis initiation and elongation^7^. In addition, NS5 proteins have been shown to inhibit the type I interferon (IFN) signalling to evade antiviral defence in the host^8^,^9^,^10^,^11^,^12^,^13^,^14^,^15^,^16^. The essential role of NS5 in viral replication and immunosuppression makes it an ideal target for antivirals^17^. However, the molecular mechanism underlying the enzymatic action of NS5 remains poorly understood. Thus far, crystal structures of full-length NS5 proteins have only been reported for JEV and DENV3 (refs 18, 19). Intriguingly, despite with ∼65% sequence identity (Supplementary Fig. 1), JEV NS5 and DENV3 NS5 adopt different orientations between the MTase and RdRp domains^18^,^19^, raising the question of how the MTase and RdRp domains of NS5 cooperate during RNA replication and capping.

To illuminate the structure and mechanism of NS5 proteins, and, more importantly, to explore potential druggable sites for ZIKV, we determined the crystal structure of full-length ZIKV NS5 in complex with *S*-adenosyl-L-homocysteine (SAH), by-product of cofactor *S*-adenosyl-L-methionine at 3.3 Å resolution. Our structural analysis reveals that ZIKV NS5 folds into one of the two alternative conformations of flavivirus NS5 proteins, providing functional implication for the conformational dynamics of flavivirus NS5 proteins. We further verify the enzymatic activity of ZIKV NS5 through a *de novo* RdRp assay using a subgenomic ZIKV RNA as template. Finally, we show that the structure of ZIKV NS5 provides a framework for future development of novel antivirals against ZIKV infection.

## Results

### Overall structure of ZIKV NS5

**Figure 1:**
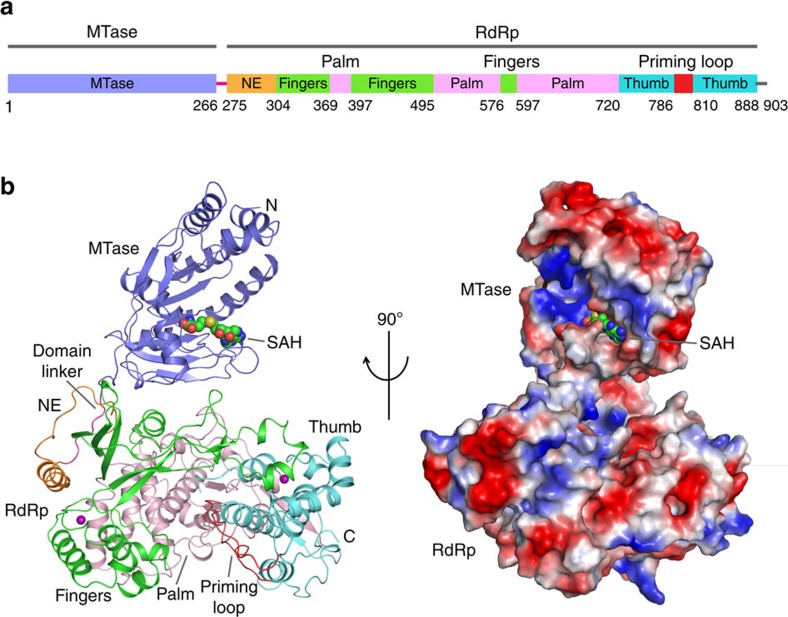
Structural overview of ZIKV NS5. (**a**) Colour-coded domain architecture of ZIKV NS5. (**b**) Orthogonal views of ribbon (left) and electrostatic surface (right) representations of ZIKV NS5. The MTase domain, the N-terminal extension, palm, fingers, priming loop and thumb of the RdRp domain, and the interdomain linker are coloured in slate, orange, pink, green, red, light blue and magenta, respectively. Zinc ions (purple) and SAH are shown in sphere representation.

We were able to trace nearly the entire sequence of ZIKV NS5 (Fig. 1a), except for the first five N-terminal residues, residues 747–748 in the RdRp domain, and sixteen residues at the C terminus. The structure reveals an N-terminal classic *S*-adenosyl-L-methionine-dependent MTase domain situated on top of a C-terminal RdRp domain. The MTase domain is dominated by a Rossmann fold, with a seven-stranded β-sheet sandwiched by two α-helices from one side and another α-helix from the other side. Near to the catalytic site, the SAH molecule is surrounded by a set of conserved residues (Supplementary Fig. 2). In addition, the N-terminal extension sequence (α1–α4 in Supplementary Fig. 1) of the MTase domain pairs with its C-terminal extension sequence (α8–β9 in Supplementary Fig. 1) to form a helix bundle and an antiparallel two-stranded β-sheet, adding another structural layer onto the Rossmann fold (Fig. 1b). As with other viral RdRps^20^, the ZIKV NS5 RdRp adopts a capped right-hand structure with the Palm, Fingers and Thumb subdomains and a priming sequence poised to receive RNA substrates (Fig. 1b). The RdRp domain also harbours two zinc ions, as observed for the NS5 proteins of JEV and DENV3 (refs 18, 19).The associations of the MTase domain with the RdRp domain does not involve extensive interdomain contacts, leading to a modest buried surface area of ∼1,400 Å^2^. In fact, structural superposition of the MTase domain in full-length NS5 and the recently reported domain alone^21^ gives a root-mean-square deviation (RMSD) of 0.42 Å over 242 Cα atoms, indicating that the MTase–RdRp association does not lead to considerable conformational change of the MTase domain.

### Structural comparison with other flavivirus NS5 proteins

**Figure 2:**
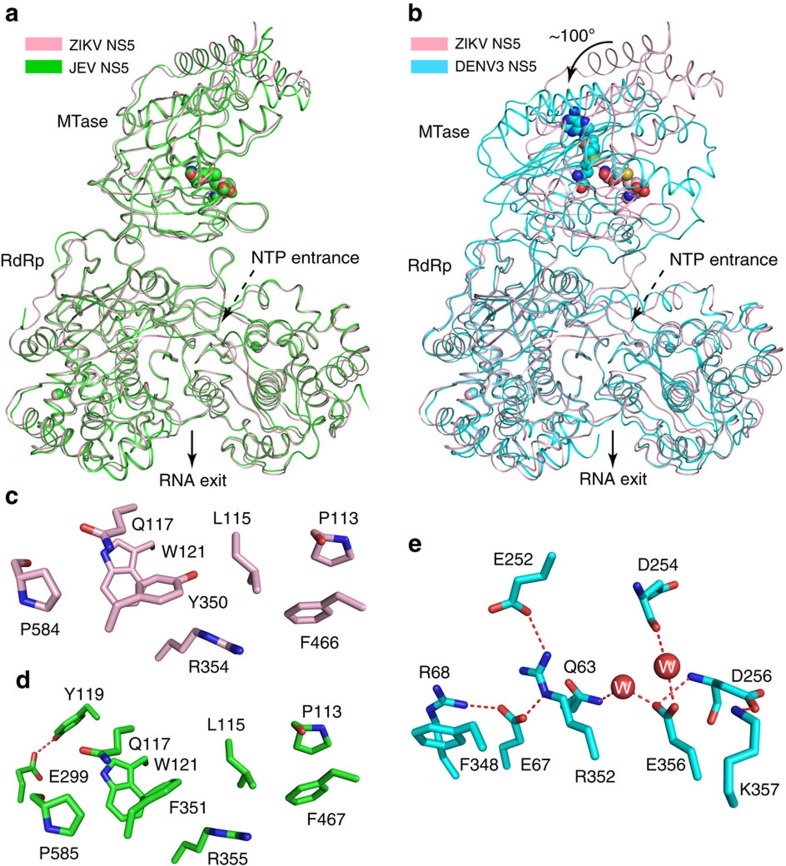
Structural comparison of NS5 proteins from ZIKV and two other flaviviruses. Structural superposition of ZIKV NS5 with (**a**) JEV NS5 (PDB 4K6M) and (**b**) DENV3 NS5 (PDB 4V0Q). Alignment of the RdRp domains of ZIKV NS5 and DENV3 NS5 leads to a ∼100° change in orientation between the MTase and RdRp domains. The NTP entrance and RNA exit sites are labelled. (**c**–**e**) The MTase–RdRp domain interactions of (**c**) ZIKV NS5, (**d**) JEV NS5 and (**e**) DENV3 NS5.

ZIKV NS5 shares ∼68% and ∼66% sequence identity, respectively, with its JEV and DENV3 counterparts (Supplementary Fig. 1). However, these NS5 homologues appear to antagonize the IFN signalling through different mechanisms: JEV NS5 suppresses IFN signalling likely through blocking phosphorylation of the IFN signalling components^13^, whereas DENV3 NS5 and ZIKV NS5 inhibit the IFN signalling through promoting protein degradation of signal transducer and activator of transcription 2 in an E3 ubiquitin ligase UBR4-dependent or -independent manner^10^,^16^. Along the line, we compared the structure of ZIKV NS5 with those of JEV NS5 and DENV3 NS5 (Fig. 2a,b). Remarkably, ZIKV NS5 superimposes well with JEV NS5, with an RMSD of 0.63 Å over 872 Cα atoms (Fig. 2a). In particular, the MTase–RdRp associations of ZIKV NS5 and JEV NS5 are both mediated by the same set of van de Waals contacts, involving the C-terminal extension of the MTase domain (P113, L115, Q117 and W121), and the Index, Ring and Middle fingers of the RdRp domain (Y350, R354, F466 and P584 in ZIKV NS5) (Fig. 2c,d). Subtle structural divergence between ZIKV NS5 and JEV NS5 was mainly observed for the N- and C-terminal extension of the MTase domain, the MTase-RdRp domain linker, and a segment in the Palm subdomain (residues E632-G653 in ZIKV NS5) (Supplementary Fig. 3a). Note that these regions have previously been linked to NS5-mediated immunosuppression^13^,^14^. Therefore, such structural divergence may underlie the distinct mechanisms of ZIKV NS5 and JEV NS5 in IFN antagonism. By contrast, structural superposition of ZIKV NS5 and DENV3 NS5 gives a RMSD of 6.06 Å over 844 Cα atoms, attributed in large part to the difference in the relative orientation between the MTase and RdRp domains (Fig. 2b). Unlike the structure of ZIKV NS5 in which the MTase domain sits on the back of the RdRp domain, the MTase domain of DENV3 NS5 approaches towards the front of the RdRp domain, resulting in a more compact conformation (Fig. 2b). Distinct from those of ZIKV NS5 (Fig. 2c,d) and JEV NS5, the MTase–RdRp association of DENV3 is mediated by a set of hydrogen bonding, cation-π and electrostatic interactions between the N- (Q63, E67 and R68) and C-terminal extensions (E252 and D254) of the MTase domain and the Index finger (F348, R352, E356 and K357) of the RdRp domain (Fig. 2e). These interactions of DENV3 NS5 appear to draw the MTase domain towards the NTP entrance of the RdRp domain, resulting in a ∼100° rotation of the MTase domain in relation to the RdRp domain (Fig. 2b). Another prominent structural difference between ZIKV/JEV NS5 and DENV3 NS5 arises from the substrate binding motifs of RdRp, including motif F in the Ring finger and motif G in the Pinky finger^22^,^23^ (Fig. 2b). The conformations of these two motifs appear to be stabilized by the MTase–Ring finger association of ZIKV/JEV NS5, but become disordered in DENV3 NS5 due to the loss of the corresponding interactions (Supplementary Fig. 3b).

The fact that the structure of ZIKV NS5 exhibits an extended domain conformation similar to that of JEV NS5, but differently from that of DENV3 NS5, raises a question on the functional implication of these two conformational states of NS5 proteins. On one hand, it is likely that the structures of ZIKV/JEV NS5 diverge from that of DENV3 NS5 through adaptive mutations of specific regions (for example, domain linker) during evolution, as proposed previously^24^. On the other hand, the high sequence conservation of both domain interfaces (Supplementary Fig. 1) strongly argues that the structures of ZIKV/JEV NS5 and DENV3 NS5 represent two alternative conformations of NS5 that may coexist in solution. Consistently, previous small-angle X-ray scattering analysis suggested the presence of a heterogeneous conformational ensemble of DENV3 NS5 in solution^24^, and mutations at the two alternative domain interfaces lead to compromised methyltransferase activity or viral replication function of DENV3 NS5 (ref. 19). Additional biochemical and cellular analyses are required to reveal the functional implication of these two alternative conformations of flavivirus NS5 proteins.

### *De novo* RdRp assay of ZIKV NS5

**Figure 3:**
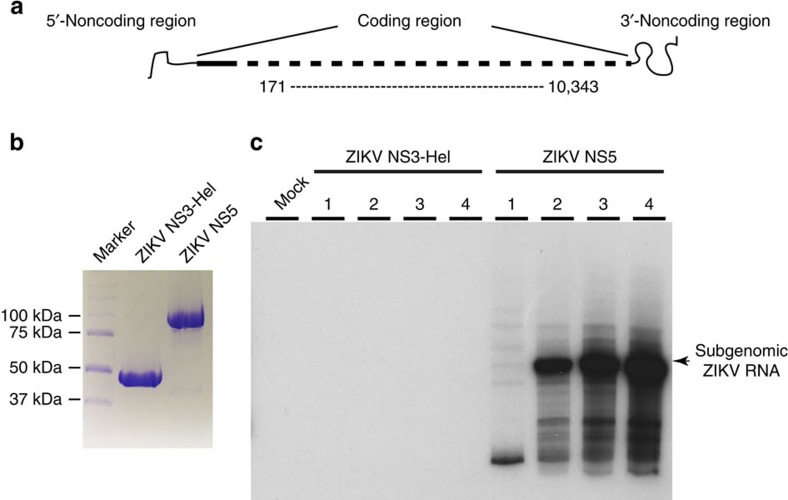
*De novo* RNA synthesis by ZIKV NS5 protein. (**a**) The subgenomic ZIKV RNA contains an internal deletion from nucleotides 171 to 10,343 (GenBank accession no. KU963573.2). (**b**) SDS–polyacrylamide gel electrophoresis analysis of purified ZIKV NS5 and ZIKV NS3-Hel. (**c**) ZIKV *de novo* RNA replication assay. The subgenomic ZIKV RNA was incubated with recombinant ZIKV NS5 protein, ZIKV NS3-Hel or alone (mock). The relative amount of ^32^P-labelled RNA product is displayed in the autoradiograph of the PAGE gel. The reactions containing recombinant proteins were divided into four groups. Group1 was incubated at 23 °C for 30 min. Groups 2, 3 and 4 were incubated at 33 °C for 30, 60 or 120 min, respectively (Supplementary Fig. 6).

To confirm that the ZIKV NS5 protein used for our structural study represents an active enzyme, we performed a *de novo* RdRp assay for ZIKV NS5 on a subgenomic ZIKV RNA template (Fig. 3a), using the recombinant ZIKV NS3 helicase domain (NS3-Hel) (Fig. 3b and Supplementary Fig. 4) as a negative control. We observed that the presence of ZIKV NS5 led to a time-dependent increase in the replication of the subgenomic ZIKV RNA at 33 °C (Fig. 3c and Supplementary Fig. 5). However, the reaction product became dominated by a shorter RNA at 23 °C, possibly due to early termination of the replication (Fig. 3c). On the other hand, the presence of ZIKV NS3-Hel failed to yield any RNA product (Fig. 3c). Together, these data not only confirm that the ZIKV NS5 protein used for the structural study is enzymatically active but also provide a basis for further functional characterization of ZIKV NS5.

### Identification of potential inhibitor-binding sites

**Figure 4:**
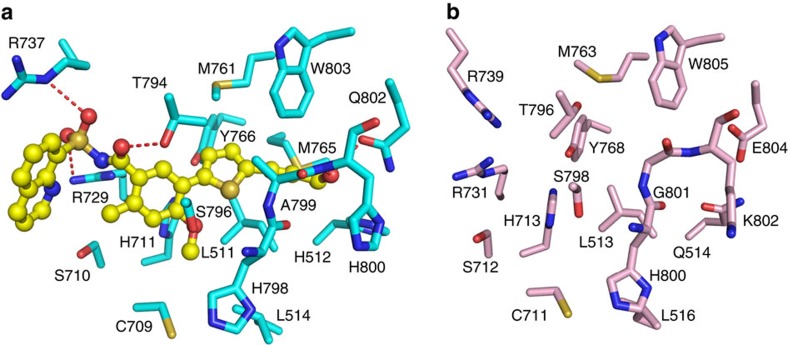
Identification of a potential inhibitor-binding site in ZIKV NS5. (**a**) Binding of a small-molecule inhibitor of DENV3 NS5 at its ‘N pocket'. The residues of DENV3 NS5 and small-molecule inhibitor are shown in blue and yellow sticks, respectively. The hydrogen bonding interactions are depicted as dashed lines. (**b**) The residues of ZIKV NS5 corresponding to the inhibitor binding site of DENV3 NS5 are shown in pink sticks.

Finally, we asked whether the structure of ZIKV NS5 permits us to identify potential inhibitor-binding sites for its enzymatic inhibition. A previous study, through fragment-based crystallography method, identified a pocket near to the active site of the DENV3 RdRp domain, termed ‘N pocket', which binds to a small-molecule that inhibits DENV3 NS5-mediated RNA initiation and elongation (Fig. 4a)^25^. Detailed analysis of this inhibitor-binding site revealed that the critical residues for the inhibitor binding are also conserved in ZIKV NS5, arranged in a similar structural environment (Fig. 4b); therefore, suggesting that the same compound may also be inhibitory to the enzymatic activity of ZIKV NS5. Further enzymatic analysis is needed to test the possibility of applying this DENV3 inhibitor to suppress the activity of ZIKV NS5.

## Discussion

Our structural study of ZIKV NS5 sheds light onto the conformational dynamics and functional regulation of ZIKV NS5. The observation that ZIKV NS5 adopts one of the two defined conformations of flavivirus NS5 proteins provides first evidence on the conservation of domain conformation within NS5 proteins, which may be required for their functional regulation. To confirm the enzymatic activity of the recombinant ZIKV NS5 protein, we developed a *de novo* RdRp assay of ZIKV NS5 on a subgenomic ZIKV RNA template, which provides basis for future mechanistic characterization of ZIKV NS5. Furthermore, we identified that the small molecular inhibitor-binding site of DENV3 NS5 is structurally conserved in ZIKV NS5, thereby revealing a potential mechanism for functional inhibition of ZIKV NS5. This study provides a foundation for future dissection of the functional coupling between the MTase and RdRp domains and a framework for the design of novel inhibitors against ZIKV infection.

## Methods

### Expression and purification of ZIKV NS5 and NS3 helicase

The DNA sequence encoding full-length ZIKV NS5 or ZIKV NS3-Hel (residues 171–617) was amplified from the cDNA of ZIKV/*Macaca mulatta*/UGA/MR-766/1947 and inserted into a modified pRSFDuet-1 vector (Novagen) (see Supplementary Table 1 for primer sequences), in which the NS5 or NS3-Hel gene was preceded by an N-terminal His_6_-SUMO tag and ULP1 (ubiquitin-like protease 1) cleavage site. The obtained plasmids were then transformed into BL21 (DE3) RIL cell strain (Agilent Technologies) for expression. The cells were first grown at 37 °C and then shifted to room temperature when *A*_600_ reached 1.0, followed by the addition of 0.4 mM isopropyl β-D-galactoside for induction. After another 18 h of cell growth, the cells were collected and the His_6_-SUMO-tagged ZIKV NS5 or ZIKV NS3-Hel was purified using a Ni-NTA affinity column. ZIKV NS5 was further purified on a Phenyl Sepharose column (GE Healthcare) for separation from degraded protein products, followed by removal of the His_6_-SUMO tag through ULP1 cleavage and size-exclusion chromatography on a Superdex 200 16/600 column (GE Healthcare) pre-equilibrated in buffer containing 25 mM Tris, pH 7.5, 500 mM NaCl, 5 mM DTT (dithiothreitol) and 5% glycerol. The ZIKV NS3-Hel fusion protein was first subject to ULP1 cleavage, and subsequently purified on a Phenyl Sepherase column and a Superdex 200 16/600 column pre-equilibrated in buffer containing 25 mM Tris, pH 7.5, 250 mM NaCl, 5 mM DTT and 5% glycerol. SDS–polyacrylamide gel electrophoresis analysis indicated that the purities of NS5 and NS3-Hel proteins were >95% and >90%, respectively. Protein solution of purified NS5 and NS3-Hel, with concentrations of ∼20 and ∼70 mg ml^−1^, respectively, were stored at −80 °C.

### Crystallization and X-ray data collection

Full-length ZIKV NS5 was mixed with SAH and GTP in a 1:3:3 molar ratio for complex formation. Initial crystallization conditions were identified through sparse-matrix screens (Hampton Research Inc.). The crystals were subsequently reproduced by hanging-drop vapour diffusion method at 4 °C, from drops mixed from 1 μl of ZIKV NS5 and 1 μl of precipitant solution (0.7–0.9 M lithium sulfate, 0.1 M MES, pH 6–7). Crystals were soaked for 1 min in a cryoprotectant solution, comprising of crystallization buffer and 20% glycerol, before flash frozen in liquid nitrogen.

The X-ray diffraction data for ZIKV NS5 were collected on the BL 5.0.3 beamline at the Advanced Light Source, Lawrence Berkeley National Laboratory. The diffraction data were indexed, integrated and scaled using the HKL2000 program^26^. The structure was solved using the molecular replacement method in PHASER^27^, with the structure of Japanese encephalitis virus NS5 (PDB ID: 4K6M) as search model. The resulting electron density revealed that there are two molecules of ZIKV NS5 in each asymmetric unit. Despite being present in the crystallization mixture, GTP molecules were not modelled, presumably due to low occupancy under the crystallization condition. The structure of ZIKV NS5 was improved by iterative model building and refinement with Coot^28^ and PHENIX^29^ software packages. The same R-free test set was used throughout the refinement. The statistics for data collection and structural refinement of ZIKV NS5 is summarized in Table 1.

### *De novo* RdRp assay

For ZIKV, the *de novo* RdRp reaction (20 μl) contained 50 mM Tris (pH 8.0), 10 mM NaCl, 5 mM MgCl_2_, 2 mM MnCl_2_, 10 mM DTT, 0.5 mM ATP, 0.5 mM UTP, 0.5 mM GTP, 5 μM CTP, 15 μCi of [α-^32^P] (10 μCi μl^−1^, 3,000 Ci mmol^−1^; Perkin-Elmer), 1 μg of RNA template and 2 μg of ZIKV NS5 protein or ZIKV NS3-Hel. The RNA template was *in vitro* transcribed from a PCR product using T7 polymerase (New England BioLabs). The PCR product contained a T7 promoter, followed by a cDNA fragment representing a ZIKV subgenome with deletion of nucleotides 171–10,343 (GenBank accession no. KU963573.2). The *de novo* RdRp reaction mixtures were incubated at 23 °C for 30 min, or 33 °C for 30, 60 and 120 min. The final reactions were further extracted with phenol–chloroform and precipitated with isopropanol. The RNA pellet was dissolved in 20 μl of 1 × denaturing gel loading dye, and loaded onto a 10% denaturing polyacrylamide gel with 7 M urea. ^32^P-labelled RNA results were detected via the autoradiograph of the PAGE gel.

### Data availability

Coordinates and structure factors for ZIKV NS5–SAH complexes have been deposited in the Protein Data Bank under accession code 5TMH. The PDB accession codes 5TMH, 5JJR, 4K6M and 4V0Q and GenBank entry KU963573.2 were used in this study. All other data are available from the corresponding authors on reasonable request.

## Additional information

**How to cite this article:** Wang, B. *et al*. The structure of Zika virus NS5 reveals a conserved domain conformation. *Nat. Commun.* **8,** 14763 doi: 10.1038/ncomms14763 (2017).

**Publisher's note**: Springer Nature remains neutral with regard to jurisdictional claims in published maps and institutional affiliations.

## Supplementary Material

Supplementary Information Supplementary Figures and Supplementary Table.

## Figures and Tables

**Table 1:** Data collection and refinement statistics.

	**ZIKV NS5**
*Data collection*	
Space group	*P*2 2_1_ 2_1_
Cell dimensions	
*a*, *b*, *c* (Å)	95.1, 136.5, 197.0
*α*, *β*, *γ* (°)	90, 90, 90
Wavelength	0.9764
Resolution (Å)	50.00–3.30 (3.42–3.30)*
*R*_sym_ or *R*_merge_	31.3 (83.5)
*I*/*σI*	5.3 (1.7)
Completeness (%)	99.4 (97.1)
Redundancy	6.2 (5.7)
CC_1/2_	0.979 (0.748)
	
*Refinement*	
Resolution (Å)	48.3–3.28 (3.36–3.28)
No. of reflections	39,405 (3,511)
*R*_work_/*R*_free_	0.262/0.293 (0.344/0.405)
No. of atoms	
Protein	13,391
Ligand	38
*B* factors	
Protein	54.12
Ligand	68.85
r.m.s.d.	
Bond lengths (Å)	0.002
Bond angles (°)	0.55

^*^Values within parentheses are for highest-resolution shell.
