# Supplementary material for: The structure of Zika virus NS5 reveals a conserved domain conformation
Source: Nat Commun. 2017 Mar 27;8:14763. doi: 10.1038/ncomms14763 (PMC5378951; doi:10.1038/ncomms14763)
Supplement: Supplementary Information — Supplementary Figures and Supplementary Table. [file ncomms14763-s1.pdf]

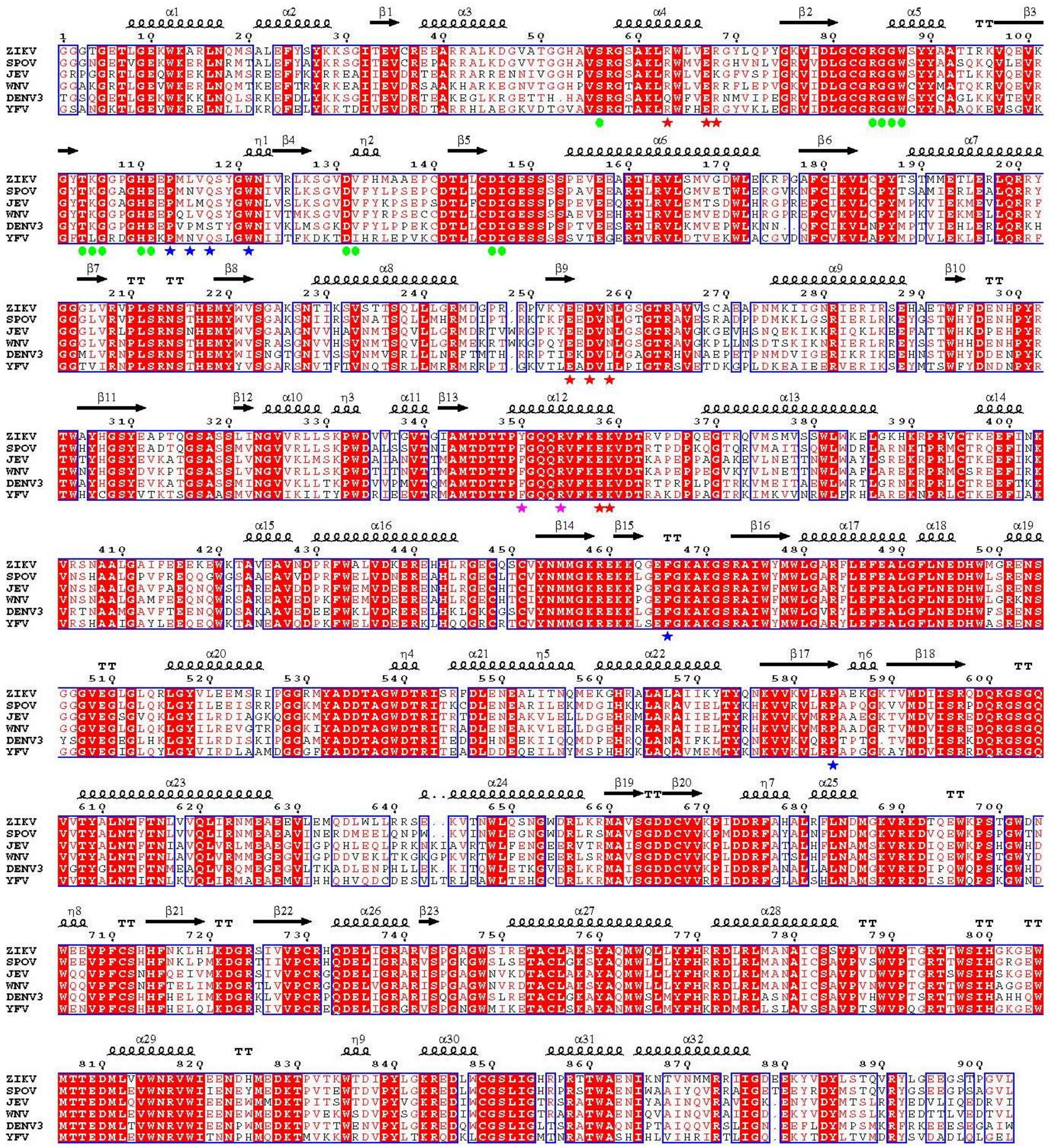

Supplementary Figure 1. **Structure-based sequence alignment of selected flavivirus NS5 proteins**. Identical or similar residues are colored in red. Completely conserved residues are colored in white and highlighted in red. Secondary structure elements are shown above the aligned sequences. Below the aligned sequences: the residues mediating SAH binding, inter-domain contact of ZIKV/JEV NS5, and inter-domain contact of DENV3 NS5 are marked by green spheres, blue asterisks and red asterisks, respectively. Note that the residues corresponding to Y350 and R354 of ZIKV NS5 (magenta asterisks) are involved in the domain interactions of both ZIKV/JEV NS5 and DENV3 NS5.

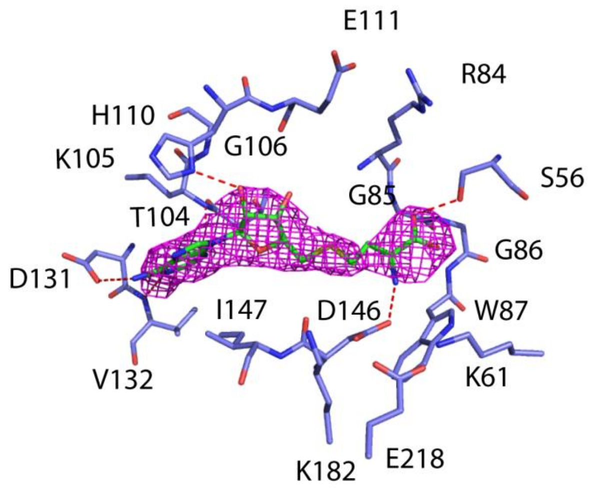

Supplementary Figure 2. **Stereo view of SAH binding.** F_O-F_C omit map of SAH is colored in magenta and contoured at 3σ level. ZIKV NS5 residues and the bound SAH molecule are shown in blue and green sticks, respectively. The hydrogen bonding interactions are depicted as dashed lines. The catalytic tetrad (K61-D146-K182-E217) is in close proximity with the SAH molecule.

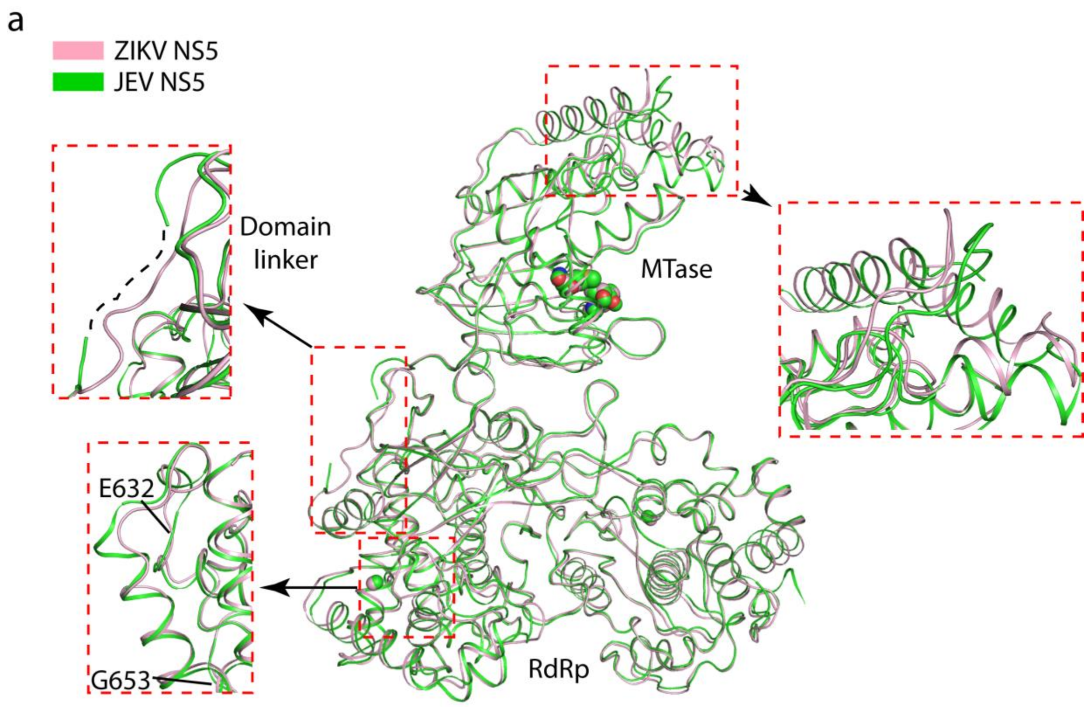

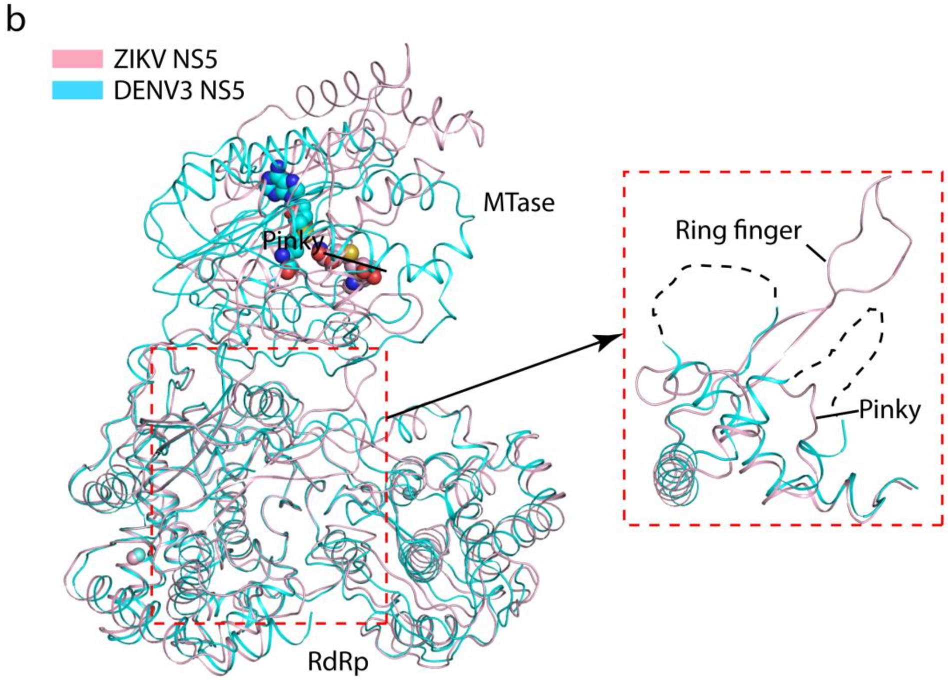

Supplementary Figure 3. **Structural comparison ZIKV NS5 with JEV NS5 and DENV3 NS5.** (a) Structural alignment of ZIKV NS5 and JEV NS5 (PDB 4K6M), with structural divergent regions shown in expanded view. (b) Structural alignment of ZIKV NS5 and DENV3 NS5 (PDB 4V0Q), with structural divergent regions shown in expanded view. The disordered loops in (a) and (b) are shown as dashed lines.

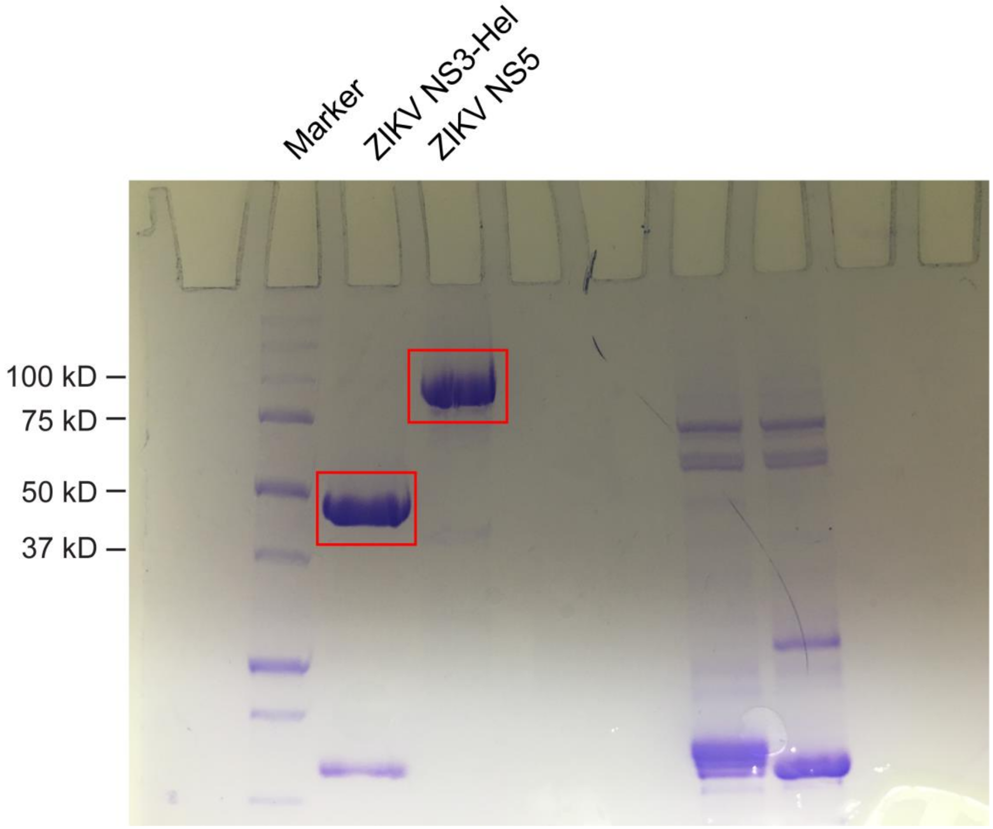

Supplementary Figure 4. **Uncropped SDS-PAGE of ZIKV NS3-Hel and NS5**. The gel bands for ZIKV NS3-Hel and ZIKV NS5 are boxed with red rectangles.

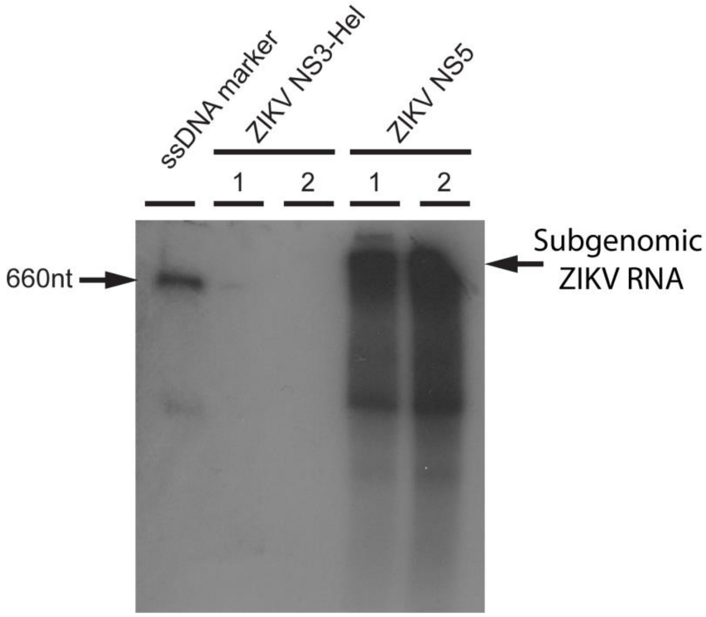

Supplementary Figure 5. *de* novo RNA replication assay of ZIKV NS5. The subgenomic ZIKV RNA was incubated with recombinant ZIKV NS5 protein or ZIKV NS3 helicase. The relative amount of ^{32}P-labelled RNA product is visualized in the autoradiograph of the PAGE gel. The reactions, divided into two groups (1 and 2), were incubated at 33°C for 60 and 120 min, respectively. The subgenomic ZIKV single-stranded DNA (ssDNA), with ^{32}P labeling at 5'-end, serves as the marker for the size of the RNA product.

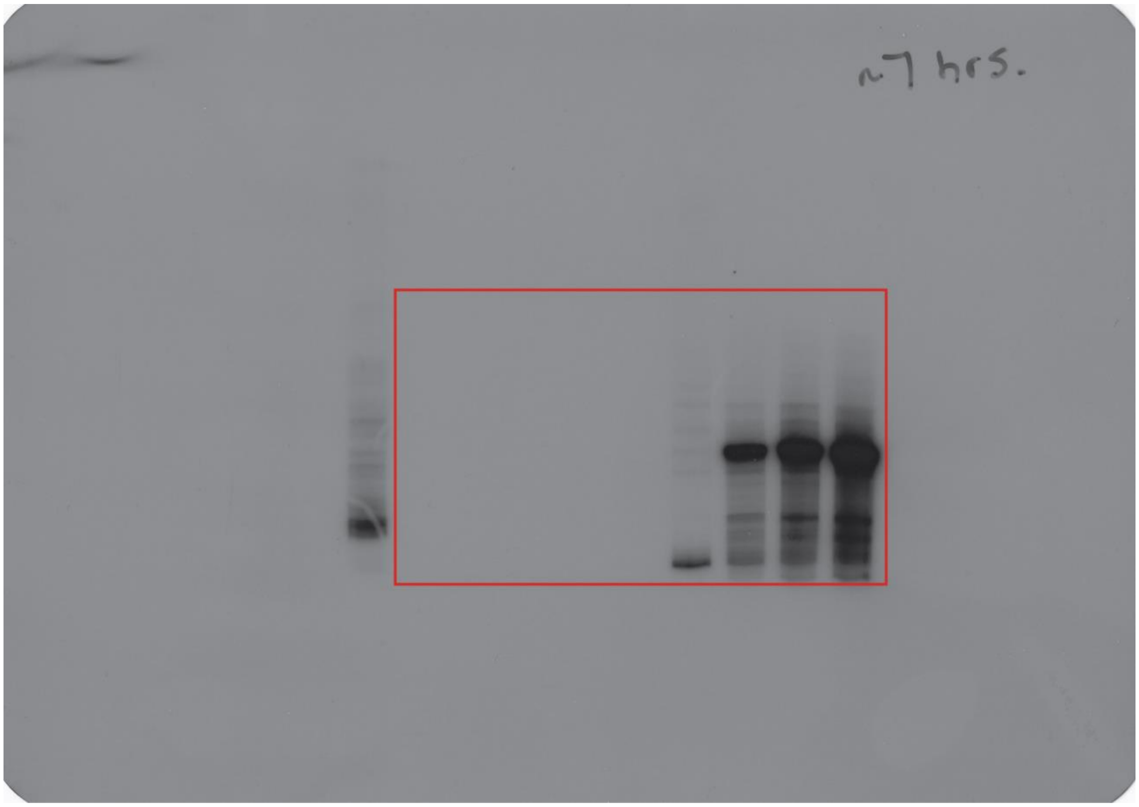

Supplementary Figure 6. Uncropped gel image for *de novo* RNA replication assay of ZIKV NS5. The gel area corresponding to the image of Figure 3c is boxed with a red rectangle.

Supplementary Table 1. Primer list for protein expression plasmids and ZIKV RNA template.

Primers	Primer sequences (5'->3')	Description
		**Generation of protein expression plasmids**
1	ACAGATTGGTGGATCCGGAG GTGGGACGGGAGAG	Forward primer for cloning of ZIKV NS5 into pRSF Duet-1 vector
2	CTTTACCAGACTCGAGTTACA ACACTCCGGGTGTGGAC	Reverse primer for cloning of ZIKV NS5 into pRSF Duet-1 vector
3	ACAGATTGGTGGATCCAGGA AGAGACTCCTGTTGAGTGC	Forward primer for cloning of ZIKV NS3 helicase into pRSF Duet-1 vector
4	CTTTACCAGACTCGAGTTATC TTTTCCCAGCGGCAAAC	Reverse primer for cloning of ZIKV NS3 helicase into pRSF Duet-1 vector
		**Generation of subgenomic ZIKV RNA template.** The subgenomic ZIKV RNA template contains two regions: The 5'- region is comprised of T7 promoter fused to ZIKV genome sequence 1-171, and the 3'-region is comprised of ZIKV genome sequence 10343-10787. These two sequences were first generated separately, followed by ligation into one ZIKV RNA template sequence.
5	TAATACGACTCACTATAGGGA GTTGTTGATCTGTGTGA	Forward primer for ZIKV RNA template 5'-region
6	GGTGCTTACAACACTCCGGG TTACACGGGCTACTCCGCG	Reverse primer for ZIKV RNA template 5'-region
7	CGCGGAGTAGCCCGTGTAAC CCGGAGTGTTGTAAGCACC	Forward primer for ZIKV RNA template 3'-region
8	TCTTGAGAATTCAGAAACCAT GGATTTCCCC	Reverse primer for ZIKV RNA template 3'-region
